# Distraction Osteogenesis for Regeneration of the Anterior Maxilla and Mandible Following a Road Traffic Accident: A Comprehensive Case Report From Saudi Arabia

**DOI:** 10.7759/cureus.105781

**Published:** 2026-03-24

**Authors:** Bishi A Algarni, Ibtihag S Elnaem, Fedaa A Mohammed Adam, Albandri M Alghris

**Affiliations:** 1 Oral and Maxillofacial Surgery, King Saud Medical City, Riyadh, SAU; 2 Oral and Maxillofacial Surgery, Riyadh Colleges of Dentistry and Pharmacy, Riyadh, SAU; 3 Oral and Maxillofacial Surgery, University of Hail College of Dentistry, Hail, SAU; 4 Oral and Maxillofacial Surgery, Consultative Clinic, Riyadh, SAU

**Keywords:** case report, distraction osteogenesis (do), oral and maxillofacial surgery, road traffic accident (rta), saudi arabia

## Abstract

Distraction osteogenesis represents a predictable and biologically driven modality for reconstructing craniofacial defects, particularly in cases involving extensive alveolar loss where traditional bone grafting alone may be insufficient. Its unique advantage lies in the concomitant expansion of both osseous and soft-tissue compartments, including neurovascular structures and attached gingiva, an outcome rarely achievable with conventional grafting approaches. We report the case of a 23-year-old male patient who sustained severe anterior maxillary and mandibular alveolar defects following a road traffic accident (RTA) in Saudi Arabia. The patient presented with loss of the anterior upper and lower dentoalveolar segments and significant maxillary impaction. Regeneration was performed using staged alveolar distraction osteogenesis with internal KLS Martin distractors (KLS Martin Group, Tuttlingen, Germany), followed by guided bone regeneration (GBR) utilizing allograft and xenograft materials and resorbable membranes, culminating in implant-supported prosthetic rehabilitation. The patient achieved excellent functional, aesthetic, and psychosocial outcomes. This case underscores the effectiveness of distraction osteogenesis as a dual-purpose strategy for hard- and soft-tissue enhancement, particularly when integrated with GBR to optimize implant-borne rehabilitation in complex anterior jaw defects.

## Introduction

Road traffic accidents (RTAs) constitute a major cause of maxillofacial trauma worldwide, often resulting in complex injuries involving the mandible, maxilla, zygomatic complex, orbit, and nasal structures [1]. Restoration of post-traumatic maxillomandibular defects presents a formidable challenge, requiring meticulous assessment of anatomical limitations, vascularity, soft-tissue quality, graft stability, and donor-site morbidity [2]. Historically, craniofacial reconstruction has relied on autogenous bone grafting supported by conventional imaging modalities and physical models. However, vertical alveolar deficiencies remain among the most challenging defects to correct due to limited vascularity and the necessity of tension-free soft-tissue closure [3-6]. Alveolar distraction osteogenesis, introduced by Chin and Toth in 1996, transformed the management of such deficiencies by exploiting the inherent capacity of bone to regenerate under controlled mechanical tension [7]. Alveolar distraction osteogenesis may be performed vertically or horizontally, enabling simultaneous augmentation of bone and periosteal soft tissues, an advantage distinct from traditional grafting [8-11]. The integration of distraction osteogenesis with guided bone regeneration (GBR) and delayed implant placement has proven reliable for restoring both form and function in cases of severe trauma or congenital deformity [12-17]. This report describes an advanced, staged reconstructive protocol combining distraction osteogenesis, GBR, and implant rehabilitation for a young male patient who sustained extensive anterior alveolar loss following an RTA.

## Case presentation

**Figure 1:**
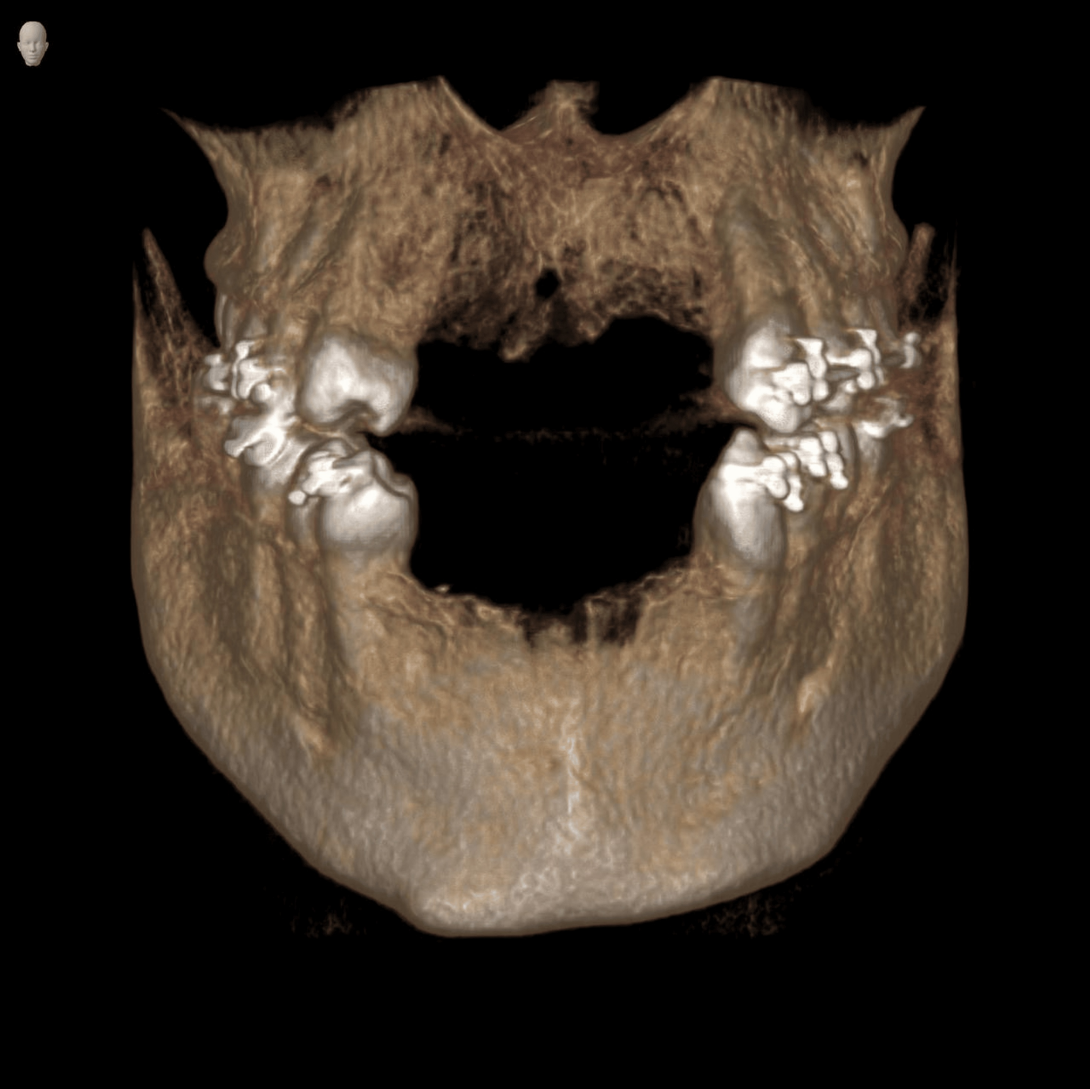
Radiographic evaluation: Three-dimensional cone-beam computed tomography (CBCT) reconstruction demonstrating the extent of anterior maxillary and mandibular bone loss.

**Figure 2:**
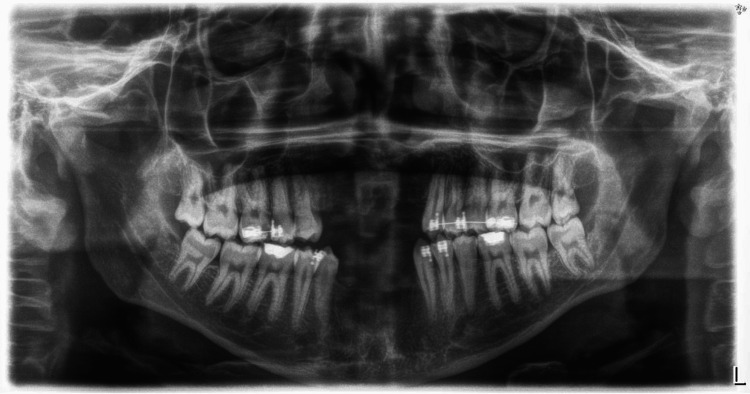
Radiographic evaluation (orthopantomogram): preoperative panoramic radiograph showing missing teeth.

A 23-year-old male patient presented to the department of oral and maxillofacial surgery with substantial post-traumatic craniofacial deformities sustained as a consequence of an RTA. The patient was systemically healthy, with no relevant prior medical or surgical history, and reported no known drug allergies. He was classified as American Society of Anesthesiologists (ASA) Physical Status Class I, indicating no systemic disease and making the patient suitable for surgical intervention under general anesthesia. The patient sustained significant orofacial trauma following an RTA. The mechanism of injury resulted in extensive dentoalveolar damage to the anterior segment of both jaws. Prior to the presentation to the oral and maxillofacial department, the patient had received the initial emergency management; however, no definitive reconstructive intervention had been undertaken. The patient reported a prolonged period of functional disability and psychological distress following the injury. The patient's chief complaints at the time of the consultation encompassed three domains. Functionally, he described severely compromised mastication, rendering him unable to consume a normal diet, alongside significantly impaired speech articulation, which adversely affected his daily communication. Aesthetically, the loss of the anterior dentoalveolar segments produced marked facial disfigurement, resulting in substantial psychosocial distress, social withdrawal, and a considerably reduced quality of life. Extra-oral assessment revealed post-traumatic changes consistent with prior dentoalveolar injury to the anterior maxilla and mandible. Soft tissue scarring was noted in the oral cavity. The facial profile demonstrated mild mid-face retraction secondary to the underlying alveolar deficiency. There was no active infection, sinus tract, or lymphadenopathy identified at the time of the examination. There is no pathology identified in the temporomandibular joint. Intraoral examination demonstrated complete loss of the anterior maxillary and mandibular dentoalveolar segments, encompassing the alveolar bone and associated dentition. The residual ridges in both anterior regions were markedly deficient in both vertical height and horizontal width, precluding conventional prosthodontic rehabilitation or standard implant placement without prior bone augmentation. There was no evidence of active inflammation or ulceration. The posterior dentition was present bilaterally with stable occlusal contact. Oral hygiene was assessed and documented as part of the pre-surgical evaluation. Radiographic evaluation was performed using cone-beam computed tomography (CBCT), as illustrated in Figures 1, 2. 

Panoramic radiography confirmed complete loss of anterior maxillary and mandibular teeth with the absence of any residual root fragments or pathological lesions. CBCT analysis provided a detailed volumetric assessment of the deficient ridges, revealing severely compromised alveolar height and width in both anterior maxillary and mandibular regions. The extent of this alveolar deficiency identified on clinical and radiographic assessment precluded reconstruction using conventional bone grafting techniques alone or single-stage implant placement. The KLS Martin Track 1 Plus distractor (KLS Martin Group, Tuttlingen, Germany) was selected for augmentation of the anterior maxillary and mandibular segments with a planned distraction distance of 20 mm in both regions. The staged treatment plan is outlined in Table 1.

**Table 1:** Staged reconstructive treatment plan

Stage	Procedure	Details	Timeline
1	Distraction osteogenesis	Vertical alveolar distraction using KLS Martin Track 1 Plus distractors (20 mm distraction); bilateral maxilla and mandible	Activation phase: 1 mm/day; consolidation: eight to 12 weeks
2	Distractor removal & bone grafting	Removal of distraction devices; autogenous/alloplastic bone grafting to augment residual defects and optimise ridge contour	Following the consolidation period
3	Implant placement	Osseointegrated dental implant placement in reconstructed anterior maxillary and mandibular ridges	Four to six months post grafting
4	Prosthetic rehabilitation	Implant-supported fixed prosthetic rehabilitation; restoration of anterior occlusion, aesthetics, and function	Following confirmed osseointegration

The expected outcome was to achieve the overarching goals of the staged reconstructive treatment plan to restore adequate alveolar bone volume to facilitate the implant placement, re-establish the normal anterior occlusal relationships, and comprehensively rehabilitate masticatory function and speech. The treatment plan aimed to restore anterior lip support, correct the post-traumatic facial profile, and achieve a result commensurate with the patient's pre-injury appearance. Distraction osteogenesis is expected to generate a minimum of 20 mm of new vertical bone height in both the anterior maxilla and mandible, producing a regenerate of adequate quality to support the implant placement. 

Treatment and outcomes

**Figure 3:**
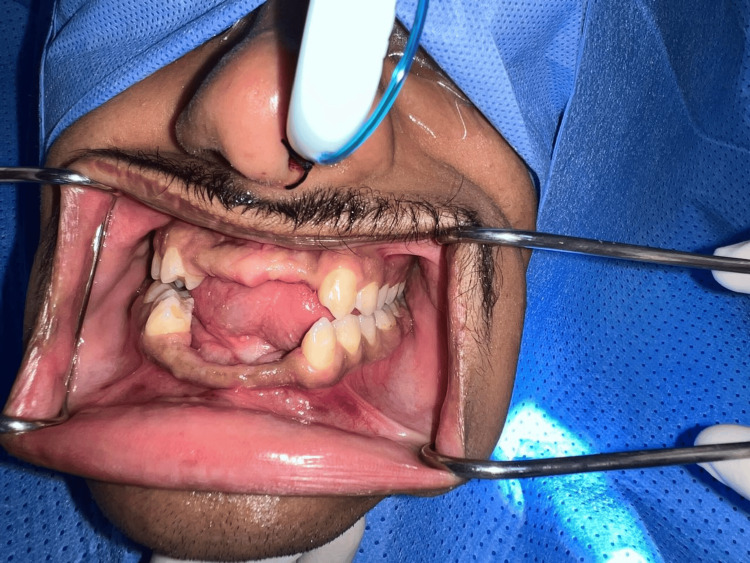
Preoperative intraoral clinical photograph illustrating severe anterior alveolar bone loss in both arches at the preoperative evaluation.

Pre-surgical preparation included comprehensive blood investigations, an anesthetic assessment, and a detailed discussion of the staged treatment plan phases with the patient. Informed consent was obtained in accordance with institutional protocols. Preoperative clinical evaluation is illustrated in Figure 3.

Stage 1: Distraction Osteogenesis

**Figure 4:**
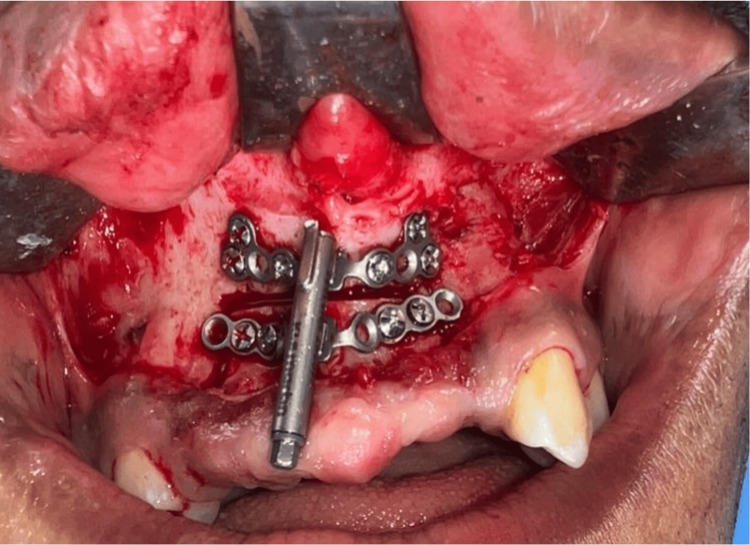
Distraction osteogenesis (maxilla) The intraoral photograph shows a maxillary distractor (KLS Martin Track 1 Plus; KLS Martin Group, Tuttlingen, Germany) in situ following an osteotomy.

Both jaws were treated under general anesthesia; for the maxilla, a mucogingival incision was performed at the junction of free and attached gingiva. The horizontal osteotomy was performed to outline the transport segment, which was carefully mobilized to verify passive movement. KLS Martin Track 1 Plus internal distractor (20 mm transport capacity) was positioned and fixed with activation parameters, and the distraction vector was confirmed intraoperatively. Soft tissues were closed using 4-0 Vicryl sutures (Ethicon, Raritan, NJ, USA). The complexity of this procedure is demonstrated in Figure 4. 

**Figure 5:**
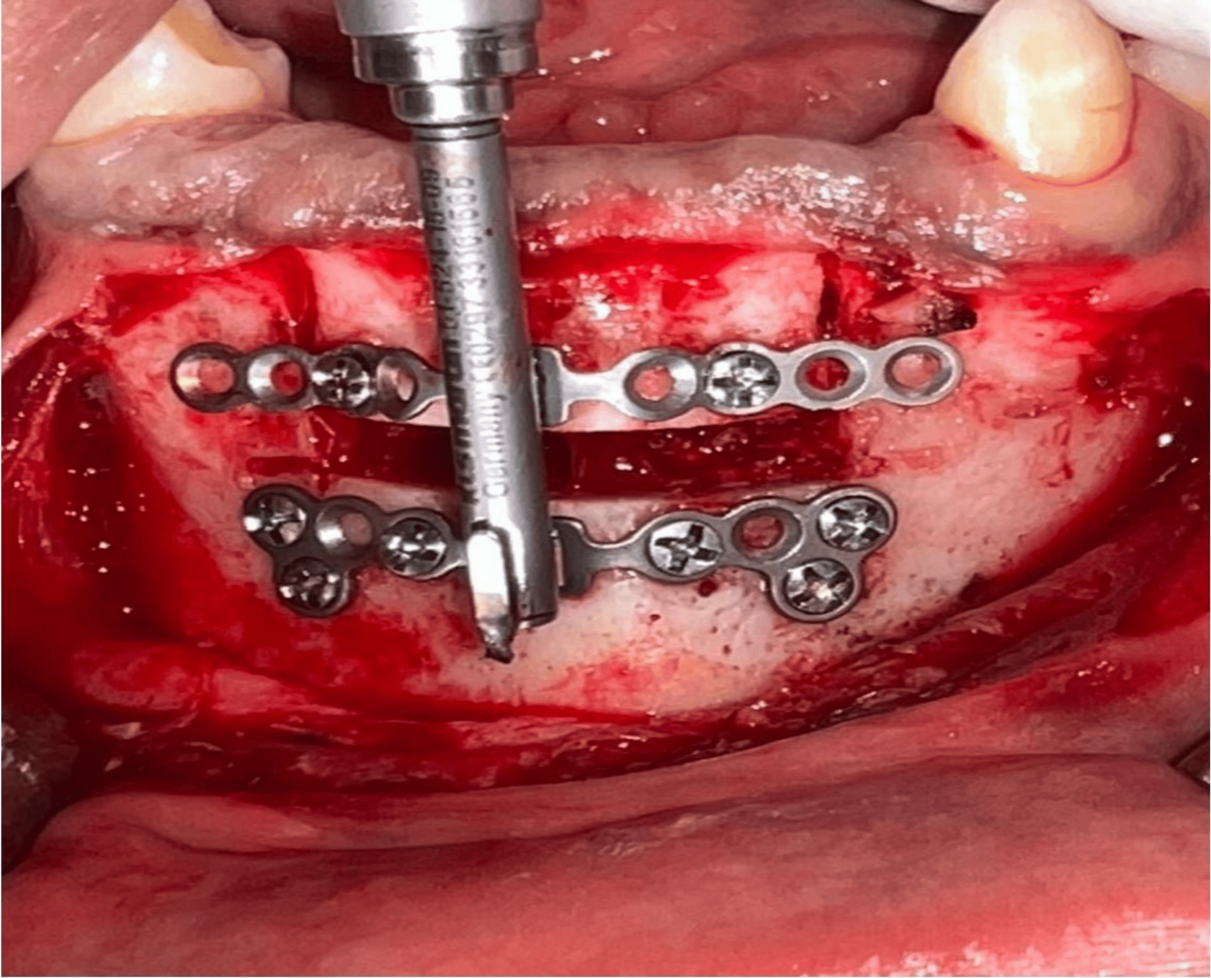
Distraction osteogenesis (mandible) Mandibular distractor in situ with mental nerve protection.

An identical procedure was performed in the mandible, with specific attention to identification and protection of the mental nerve prior to osteotomy completion and distractor placement, as illustrated in Figure 5.

**Figure 6:**
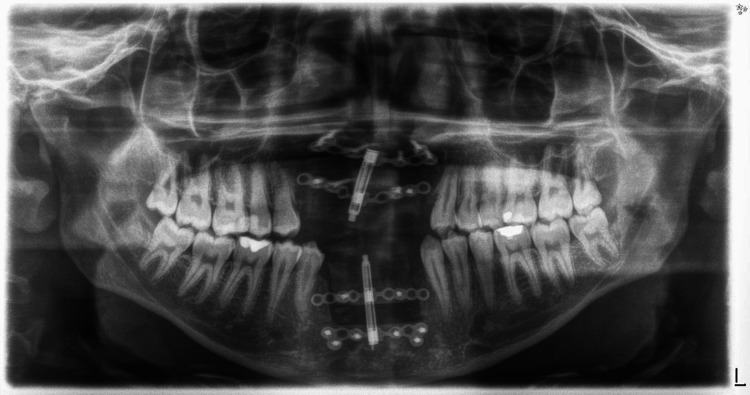
Post-distraction panoramic radiographic assessment (orthopantomogram) at 12 weeks.

**Figure 7:**
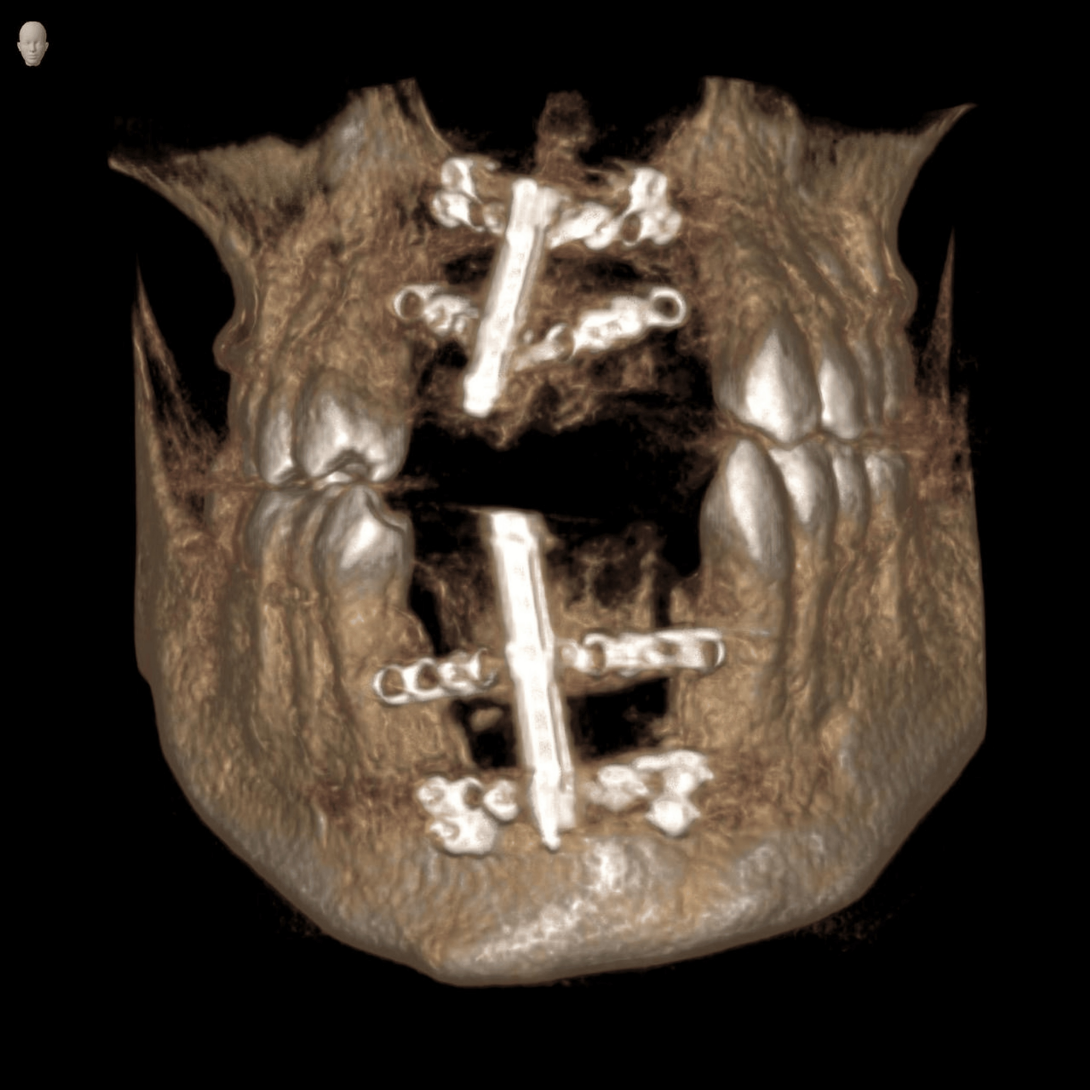
Radiographic evaluation (cone-beam computed tomography (CBCT)): three-dimensional reconstruction of the CBCT demonstrates regenerated alveolar bone in both jaws.

Following a seven-day latency period to permit initial callus formation, gradual activation was commenced at a rate of 0.9 mm per day, administered in increments of 0.33 mm every eight hours. The patient maintained a written activation log and was reviewed on a weekly basis. Analgesics, prophylactic antibiotics, and chlorhexidine 0.12% mouthwash were prescribed throughout the active distraction phase. A consolidation period of 12 weeks was observed. Successful bone regeneration was confirmed radiographically by panoramic and CBCT, as illustrated in Figures 6, 7.

Stage 2: Distractor Removal and GBR 

Following the consolidation phase, the patient was taken to the operating room under general anesthesia. Both distractors were removed via mucogingival incisions in the maxillary and mandibular regions. Intraoperative assessment revealed adequate vertical bone gain; however, horizontal ridge width remained suboptimal for implant placement. GBR was, therefore, performed concurrently: a combination of allograft and xenograft was placed over the regenerated ridges and covered with resorbable collagen membranes to enhance bone volume and density. A four-month healing period was followed by CBCT to confirm the outcome of the bone graft for implant placement.

Stage 3: Implant Placement

**Figure 8:**
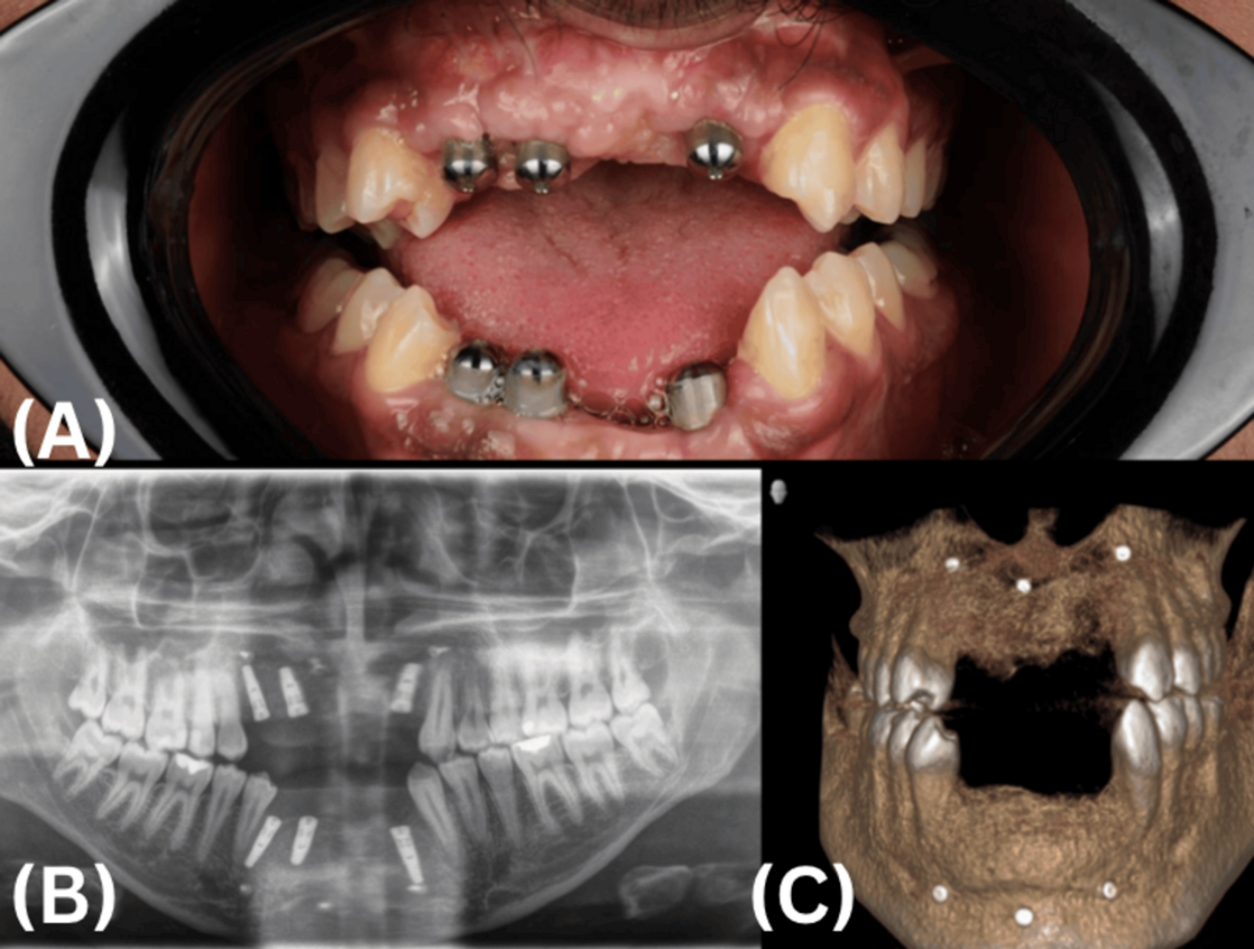
Implant placement (A) An intraoral frontal view photograph showing six implant cover screws in situ in the anterior maxilla and mandible; (B) Panoramic radiograph confirming implant position; (C) Three-dimensional reconstructive cone-beam computed tomography confirming implant position.

Following the four-month GBR consolidation period, six Biomet 3i dental implants (Biomet 3i LLC, Palm Beach Gardens, FL, USA) were placed under local anesthesia: three in the anterior maxilla and three in the anterior mandible, positioned according to a prosthetically driven surgical guide. A standard drilling protocol was followed, and primary stability was achieved in all sites. Wound closure was performed using a 4-0 Vicryl suture, and standard postoperative instructions were provided. The implant placement stage is showcased in Figure 8.

Stage 4: Prosthetic Rehabilitation and Outcome

**Figure 9:**
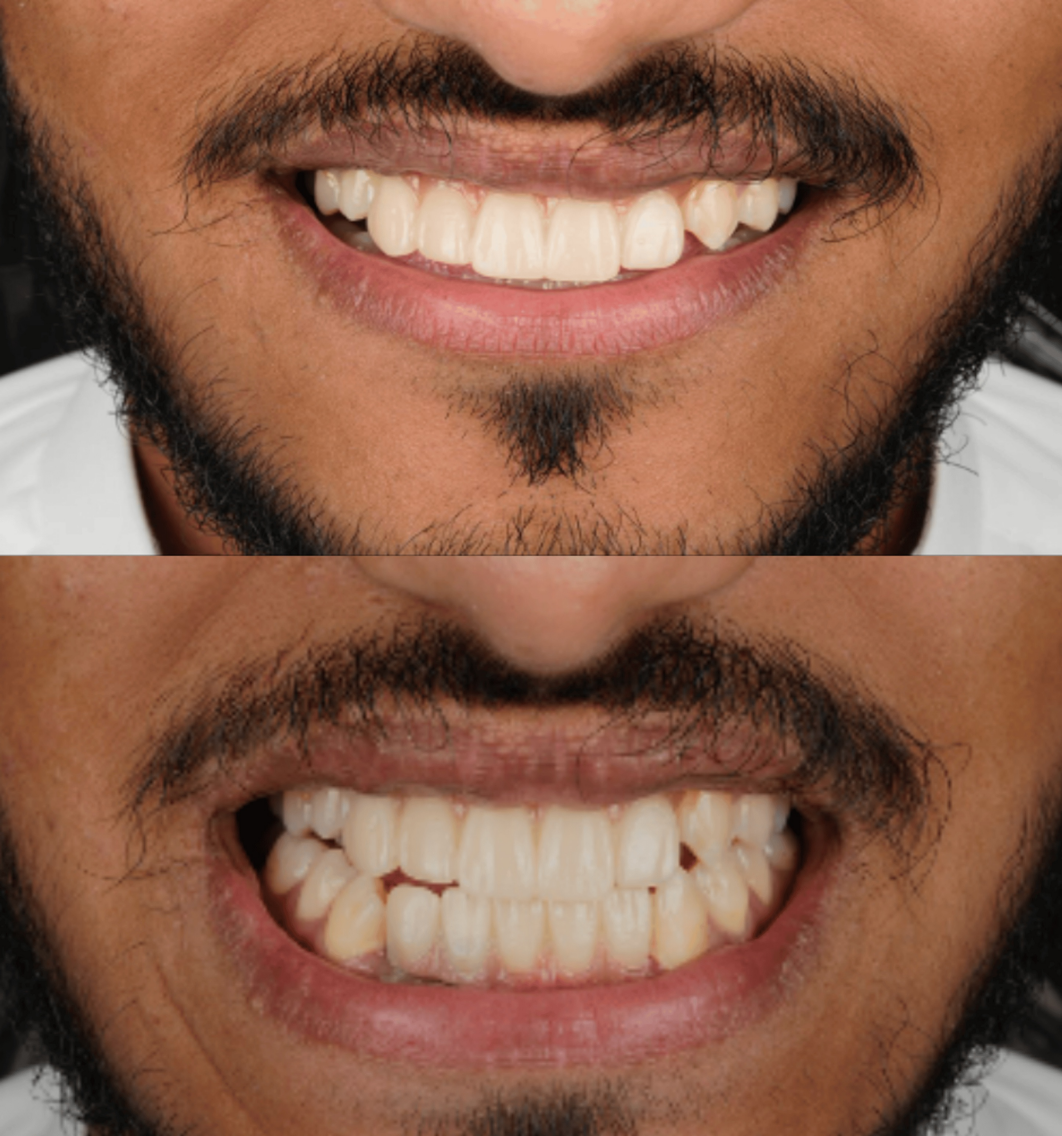
Prosthetic rehabilitation Frontal view demonstrating a fine aesthetic outcome following implant-supported crown placement, showing natural alignment, occlusion, and soft tissue integration from two angles.

Three months following implant placement, the implants were surgically uncovered, healing abutments were connected, and soft tissue contouring was achieved over a two-week maturation period. Fixed implant-supported zirconia-ceramic restorations were subsequently fabricated and delivered, restoring anterior dental form, function, aesthetics, and phonetics as shown in Figure 9.

**Figure 10:**
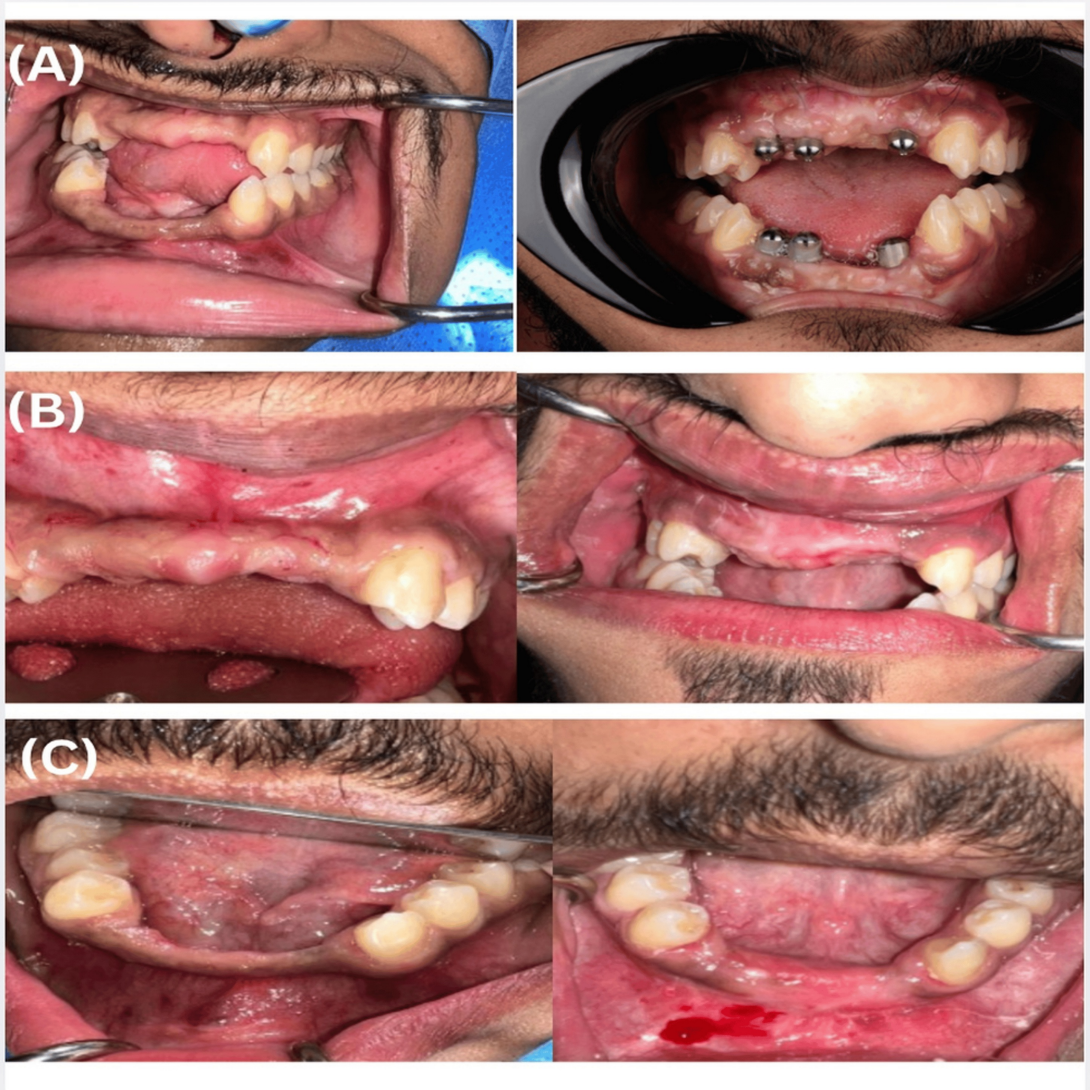
Comparative preoperative and postoperative intraoral clinical photographs For each panel, the left image represents the preoperative status and the right image represents the postoperative outcome. (A) Occlusal views of the maxillary and mandibular arches demonstrating dentition status before and after treatment. (B) Frontal intraoral views illustrating soft tissue condition and occlusal relationships before and after intervention. (C) Lateral intraoral views confirming functional and aesthetic improvement following treatment completion.

**Figure 11:**
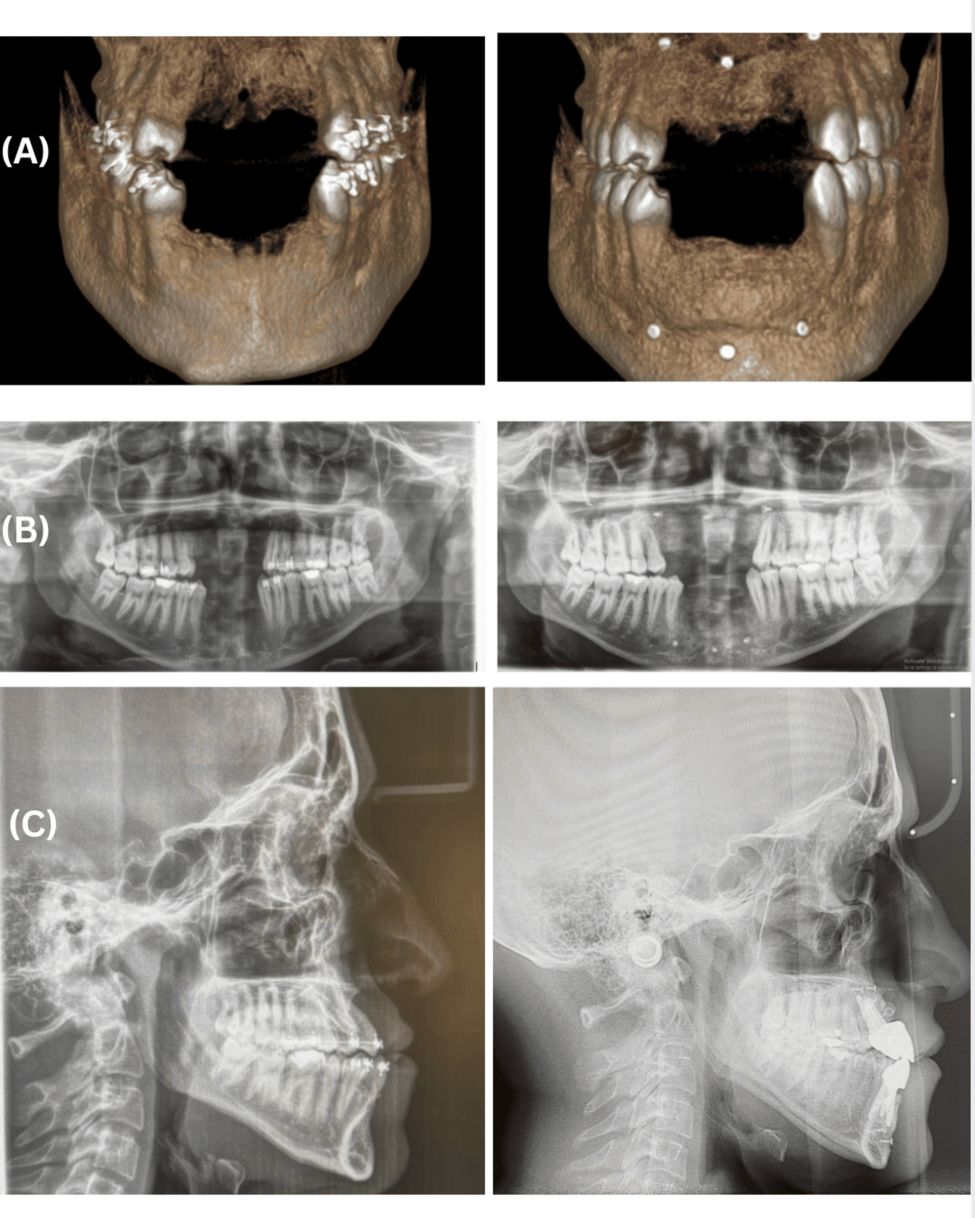
Comparative preoperative and postoperative radiographic evaluation For each panel, the left image represents the preoperative status, and the right image represents the postoperative outcome. (A) Three-dimensional cone-beam computed tomography reconstructions demonstrating skeletal and dentoalveolar morphology before and after surgical intervention. (B) Panoramic radiographs (orthopantomograms) illustrating the overall dental and osseous architecture before and after treatment. (C) Lateral cephalometric radiograph confirming skeletal and occlusal changes following surgical-orthodontic treatment completion.

The patient reported substantial improvement in quality of life, self-confidence, and social functioning. Scheduled follow-up assessments at three, six, and 12 months post-loading demonstrated stable marginal bone levels, satisfactory peri-implant soft tissue health, and complete osseointegration, with no biological or prosthetic complications recorded throughout the follow-up period. The patient's preoperative and postoperative status is depicted in Figures 10, 11.

The staged reconstructive treatment plan protocol's clinical timeline is provided in Table 2.

**Table 2:** Clinical timeline: summary of the staged reconstructive protocol GA: general anesthesia; CBCT: cone-beam computed tomography; GBR: guided bone regeneration; RTA: road traffic accident

Stage	Event/Procedure	Timing	Key Details
Preoperative	RTA: initial injury; clinical & CBCT evaluation	Day 0	Loss of anterior maxillary & mandibular dentoalveolar segments (right canine to left lateral incisor)
Stage 1	Distraction osteogenesis: maxilla & mandible	Week 1	KLS Martin Track 1 Plus distractors placed; latency period seven days; activation 0.9 mm/day (0.33 mm/8 hours)
Consolidation	Radiographic confirmation of regenerated bone	Weeks 1–12	Twelve-week consolidation phase confirmed by panoramic radiograph and CBCT
Stage 2	Distractor removal and GBR	Month 3	Devices removed under GA; allograft + xenograft with resorbable collagen membranes applied
Healing	Post-GBR healing	Months 3–7	Four-month healing; CBCT performed to confirm bone volume for implant planning
Stage 3	Implant placement	Month 7	Six Biomet 3i implants were placed (three anterior maxilla, three anterior mandible) under local anaesthesia
Osseointegration	Implant osseointegration	Months 7–10	Three-month healing; implant uncovering and healing abutment placement
Stage 4	Prosthetic rehabilitation	Month 10	Fixed implant-supported restorations delivered; function, aesthetics, and phonetics restored
Follow-up	Post-prosthetic follow-up	Months 13, 16, 22	Stable bone levels and implant integration confirmed at three, six, and 12 months post loading

## Discussion

Traumatic maxillofacial defects impose profound functional, aesthetic, and psychological burdens on young patients. Autogenous bone grafting has long been regarded as the gold standard for alveolar reconstruction; however, its limitations in this context are well established and include donor-site morbidity, unpredictable graft resorption, and insufficient soft tissue augmentation capacity [18]. These drawbacks are especially pronounced in the aesthetic zone, where an adequate volume of keratinized attached gingiva is as critical to long-term implant success as the underlying bone height. Distraction osteogenesis addresses these shortcomings through the tension-stress principle, exploiting the innate regenerative capacity of bone and adjacent soft tissue when subjected to controlled progressive distraction forces. Simultaneously generating new bone (osteogenesis) and soft tissue (histogenesis), distraction osteogenesis is uniquely suited to the correction of vertical alveolar deficiencies, defects that remain notoriously resistant to conventional grafting techniques because of poor vascularity [3-6]. These biological advantages are particularly relevant in the anterior maxilla, where soft tissue volume determines the emergence profile and long-term aesthetics of implant-supported restoration [6]. In the present case, staged vertical distraction osteogenesis successfully restored alveolar height in both jaws, generating bone and simultaneously expanding the overlying soft tissue sleeve. The secondary GBR step, combining allograft and xenograft with resorbable collagen membranes, was employed to improve horizontal ridge dimensions and bone quality prior to implant placement, an approach consistent with the PASS principle (primary closure, angiogenesis, space maintenance, stability) advocated by Wang and Boyapati [7]. This combined protocol is supported by Toledano-Serrabona et al. [17], whose systematic review confirmed favorable outcomes of distraction osteogenesis for implant rehabilitation in cases with severely deficient residual ridges. Similarly, Chiapasco et al. [11], in a multi-center prospective study, demonstrated reliable implant survival rates and stable marginal bone level following distraction osteogenesis with secondary GBR, consistent with the long-term stability observed in our patient. The distraction parameters selected in this case, a seven-day latency period and an activation rate of 0.9 mm/day, conform to well-established distraction osteogenesis protocols. Mohanty et al. [10] described comparable activation rates for vertical distraction osteogenesis, noting that rates exceeding 1.0 mm/day carry the risk of premature consolidation, while rates below 0.5 mm/day predispose to fibrous union. Faysal et al. [16] further demonstrated that a consolidation period of at least 12 weeks optimizes bone maturation and implant success rates in the distracted segment, validating the 12-week consolidation period used here. Yamauchi et al. [9], reporting on the long-term outcome of horizontal distraction osteogenesis, likewise found stable implant integration when sufficient healing was permitted before prosthetic loading. Complication avoidance was a central element of the surgical planning in the case. Mental nerve identification and preservation during mandibular distraction osteogenesis, selective use of internal distractors to reduce the risk of vector deviation, and staged implant placement after confirmed bone maturation collectively contributed to the absence of neurosensory, infectious, or prosthetic complications. Enislidis et al. [15] reported complication rates of up to 30% in alveolar distraction osteogenesis, most commonly related to vector deviation and wound dehiscence; the absence of such events in the present case reflects meticulous surgical execution and close postoperative monitoring. Collectively, the evidence and this case reinforce the position that distraction osteogenesis, when combined with secondary GBR and prosthetically guided implant placement, provides a reliable and biologically superior reconstructive pathway for young patients with extensive post-traumatic anterior jaw defects.

## Conclusions

This case demonstrates that a staged protocol incorporating vertical alveolar distraction osteogenesis, secondary guided bone regeneration, and implant-supported rehabilitation constitutes an effective and biologically sound strategy for the management of extensive post-traumatic anterior maxillomandibular defects. Distraction osteogenesis, by simultaneously regenerating hard and soft tissue through progressive tension stress, overcomes the principal limitations of conventional bone grafting in the setting of major vertical alveolar deficiency. The integration of secondary guided bone regeneration further optimized horizontal bone dimensions, providing an anatomical foundation that supported fully osseointegrated implants and stable, aesthetically satisfying fixed restorations. Long-term follow-up of distraction osteogenesis proved to be reliable and biologically driven; three, six, and 12 months post-loading confirmed the durability of both osseous and prosthetic outcomes, with marked improvement in the patient's functional capacity, facial harmony, and psychosocial well-being. This case reinforces the value of a multidisciplinary, staged approach in complex craniofacial reconstruction that contributes to the growing body of evidence supporting distraction osteogenesis as a first-line modality for large anterior jaw defects in young, otherwise healthy patients.
